# Technical optimization of spatially resolved single-cell transcriptomic datasets to study clinical liver disease

**DOI:** 10.21203/rs.3.rs-3307940/v1

**Published:** 2023-09-05

**Authors:** Brittany Rocque, Kate Guion, Pranay Singh, Sarah Bangerth, Lauren Pickard, Jashdeep Bhattacharjee, Sofia Eguizabal, Carly Weaver, Shefali Chopra, Shengmei Zhou, Rohit Kohli, Linda Sher, Burcin Ekser, Juliet A. Emamaullee

**Affiliations:** University of Southern California; University of Southern California; University of Southern California; University of Southern California; University of Southern California; Children’s Hospital Los Angeles; University of Southern California; Children’s Hospital Los Angeles; University of Southern California; Children’s Hospital Los Angeles, University of Southern California Los Angeles; Children’s Hospital Los Angeles; University of Southern California; Indiana University School of Medicine, Indiana University; University of Southern California

**Keywords:** Spatial transcriptomics, deconvolution, biliary atresia, cirrhosis, liver disease

## Abstract

Single cell and spatially resolved ‘omic’ techniques have enabled deep characterization of clinical pathologies that remain poorly understood, providing unprecedented insights into molecular mechanisms of disease. However, transcriptomic platforms are costly, limiting sample size, which increases the possibility of pre-analytical variables such as tissue processing and storage procedures impacting RNA quality and downstream analyses. Furthermore, spatial transcriptomics have not yet reached single cell resolution, leading to the development of multiple deconvolution methods to predict individual cell types within each transcriptome ‘spot’ on tissue sections. In this study, we performed spatial transcriptomics and single nucleus RNA sequencing (snRNASeq) on matched specimens from patients with either histologically normal or advanced fibrosis to establish important aspects of tissue handling, data processing, and downstream analyses of biobanked liver samples. We observed that tissue preservation technique impacts transcriptomic data, especially in fibrotic liver. Deconvolution of the spatial transcriptome using paired snRNASeq data generated a spatially resolved, single cell dataset with 24 unique liver cell phenotypes. We determined that cell-cell interactions predicted using ligand-receptor analysis of snRNASeq data poorly correlated with celullar relationships identified using spatial transcriptomics. Our study provides a framework for generating spatially resolved, single cell datasets to study gene expression and cell-cell interactions in biobanked clinical samples with advanced liver disease.

## INTRODUCTION

Biliary atresia (BA), the most common cause of end-stage liver disease in childhood, is clinically characterized as an perinatal progressive cholangiopathy affecting the bile ducts with a range of hypothesized etiologies.^1^ Arising from a variety of potential sources, including targeted epithelial injury, defective embryogenesis, environmental toxins, and viruses, the immune landscape of BA is complex. Studies have shown CD4 + Th1 cell-mediated inflammation plays a critical role in the development of biliary obstruction.^2^ Type-2 cytokines are known to induce hepatic fibrosis and cholangiocyte proliferation and have been a focal point of investigation in BA.^3^ However, changes in gene expression profiles as well as the interplay between cholangiocytes and other cell types in the liver of patients with BA remain poorly defined.

Single-cell transcriptomic analyses created from cells in suspension including single-cell RNA-seq (scRNAseq) or single nucleus RNA-seq (snRNAseq) have provided mechanistic insights into liver disease in scarce clinical specimens at a resolution that was previously not possible.^4–6^ These techniques have emerged as a valuable tool for the study of discrete cell phenotypes to enhance our understanding of disease states and identify potential therapeutic targets in liver disease. A normally functioning liver has preserved spatial architecture, including zones of discretely functioning hepatocytes, regions enriched with liver resident leukocytes, and areas designated for the production and excretion of bile, among others. Despite this intricate organization, to date, few studies have focused on the spatial aspects of liver disease pathogenesis, including processes involved in BA. MacParland et al. determined that non-inflammatory macrophage genes were localized in periportal regions while inflammatory macrophage genes were found closer to the central vein in healthy liver tissue.^7^ Coupling spatial sequencing technologies, Melum et al. found that mesenchymal, monocyte, B cell, and T-cell lineages within fibrotic regions were more prevalent than within parenchyma, suggesting that these subsets play important roles in hepatic fibrogenesis.^8^ Incorporating spatial features into single-cell transcriptomics has the potential to examine important cellular interactions within the liver, allowing for the identification of more precise mechanisms of liver disease and highlights potential future therapeutic interventions.

A variety of spatial transcriptomic platforms are in development that enable analysis of gene expression patterns across histological regions of interest.^9^ Spatial transcriptomics provide the additional benefit of describing the pathologic tissue landscape of diseases; however, best practices for the use of these techniques, particularly within the complex architecture of the liver, are unknown. Spatial transcriptomic data currently require integratation with other sequencing modalities via computational deconvolution methods to provide single-cell resolution and therefore gain novel insights into cellular features of disease processes with spatial manifestations.^10,11^ Limitations include the cost associated with transcriptomic techniques and generation of highly dimensional and complex datasets, which can limit the volume of samples that can be processed and analyzed. Moreover, pre-analytical variables such as tissue storage and processing techniques can influence sequencing outputs, which may unintentionally confound results obtained from samples that are collected and stored in the clinical setting.^12^

The purpose of this study was to perform spatial transcriptomic analysis of the human liver, including samples of histologically normal liver and BA with advanced fibrosis. First, we employed two unique tissue preservation techniques within the same samples to determine the reproducibility of spatial transcriptomic data across experimental conditions. This is relevant to investigators who work with biorepositories containing frozen liver specimens. We integrated paired snRNAseq datasets to deconvolute the spatial profile and achieve single-cell resolution for 24 different liver cell phenotypes. We evaluated the correlation between predicted cell-to-cell interactions derived from ligand-receptor analysis of snRNAseq data to spatial transcriptomic outputs from the same tissue sample to establish the reliability of ligand-receptor (L-R) analysis to reflect true spatial motifs. We then focused our analysis on the spatially resolved molecular differences between normal and BA liver with advanced fibrosis. Together, these analyses provide a reference for performing spatial transcriptomic analysis on both healthy and fibrotic human liver tissue.

## METHODS

This study was begun after obtaining approval from the Institutional Review Board at the University of Southern California (CHLA-19-00177). All research was performed in accordance with the Declaration of Helsinki, and informed consent from legal guardians (patients were under 18 years of age at enrollment) was obtained for all tissue and clinical data obtained for this study.

### Clinical Specimens:

Human fresh liver tissue specimens were collected from explants from patients undergoing liver transplant. For the purposes of this study, an area of histologically normal liver obtained from a patient undergoing transplant for hepatoblastoma was designated as ‘normal’ liver tissue while liver tissue with advanced fibrosis was obtained from a patient undergoing transplant for end-stage BA (confirmed via Hematoxylin and Eosin (H&E) staining and review by expert pathologists). Fresh liver was placed in sterile saline and transported to the lab on ice. Samples were diced to approximately 0.5 cm cubes, and portions were either snap-frozen in liquid nitrogen (SNAP) or cryopreserved in OCT blocks (OCT). These samples were stored in liquid nitrogen. On the day of sample processing for transcriptomic analysis, SNAP specimens were blocked using OCT without prior thawing. Both sets of OCT-embedded specimens were then transported on dry ice to the Molecular Genomics Core of the Norris Comprehensive Cancer Center of USC for Visium Spatial Gene Expression Assay (10X Genomics).

### Transcriptomics:

Spatial transcriptomic profiling was performed using the 10x Genomics Visium CytAssist. From the same sample block, scrolls were sectioned, and a sequential slide stained with H&E was reviewed by a liver pathologist to select regions of interest including portal, periportal, and centrivenular areas. RNA extraction from the tissue scrolls was performed using the Qiagen RNeasy kit, and RNA quality control was assessed using the Agilent BioAnalyzer. The sample block was sectioned at 10μm onto charged slides and transferred to the spatial profiling slides through the CytAssist instrument and proceeded into library preparation. Quality control of the libraries was performed on the Agilent BioAnalyzer and KAPA library quantification. Samples were sequenced on the Illumina Novaseq6000 S2 and data was packaged using the 10x Genomics SpaceRanger pipeline.

### Single nucleus RNA-seq:

Since scRNAseq needs to be performed on fresh tissue, snRNAseq was chosen instead given its ability to be performed on previously frozen samples. snRNAseq was performed using the 10x Genomics Chromium X platform with 10x Genomics 3’ gene expression kit. Cell count and viability of the nuclei were verified on the ThermoFisher Countess 3 cell counter and EVO imager microscope. Single nuclei were parsed and proceeded into library preparation following manufacturer protocol. Quality control of the libraries was performed on the Agilent BioAnalyzer and Kapa library quantification. Samples were sequenced on the Illumina Novaseq6000 SP and data was packaged using the 10x Genomics CellRanger pipeline. The snRNAseq and spatial transcriptomic samples were patient-matched to increase the reliability of results during deconvolution and analysis.

### Quality Control and data preprocessing:

Data were imported into R and converted into Seurat objects. For quality control, cells with greater than 10% mitochondrial genes as well as cells with less than 200 genes in total were excluded from the analysis. Mitochondrial, ribosomal, and heat shock protein genes were also excluded from the analysis. To preserve biological variance present in each tissue sample, “SCTransform”, which implements regularized negative binomial models, was performed on each sample individually to normalize the data and detect high-variance genes.^13^ The same quality control measures were implemented per spatial spot in the Visium data using Seurat. Any genes that were not measured in both the snRNA and Visium spatial transcriptomics were excluded from the analysis.

### Single nucleus analysis:

snRNAseq data from normal and BA livers were integrated following the Seurat data integration pipeline.^10^ Dimensionality reduction and data clustering were performed after data integration using the Seurat “FindNeighbors” and “FindClusters” functions, optimizing for the number of nuclei in each sample. Gene expression heatmaps were used to aid in cluster and cell type annotation, which resulted in 24 subclusters (a total of 13 metaclusters). Proportions of each cell type were compared across samples and statistical significance was determined using permutation testing (1,000 permutations; “scProportionTest”^14^).

### Spatial transcriptomics:

Spatial samples were integrated following the Seurat data integration pipeline.^10^ Differential gene expression (DE) was performed to identify differences in gene expression between datasets. Genes that were up- or downregulated in each comparison were identified and highlighted using a volcano plot. Pathway analysis was performed using the DE output to further compare datasets. Clustering was done on the spatial samples to annotate the spatial spots.

### Spatial deconvolution using single-nucleus data:

The annotated snRNAseq samples were used for spatial single-cell deconvolution using CellTrek (version 0.0.94).^15^ This process mapped individual annotated snRNAseq cell types to geographical locations within the tissue. To minimize false positives, specific parameters were selected, allowing each nucleus to be mapped to up to five distinct spatial spots while ensuring that each spot contained no more than five different cells. The resulting labels were visualized on the tissues.

### Correlation analysis:

Once the snRNAseq cell types were given spatial coordinates, CellTrek::scoloc was used to examine the colocalization of different cell types. This method calculates a 2D grid kernel density for each cell type and then uses Kullback-Leibler divergence to uncover global spatial patterns. Spatial relationships between different cell types where then visualized using the igraph R package (version 1.4.2).^16^

### Ligand-Receptor analysis:

The CellChat (version 1.6.1) package was used to perform L-R analysis on both the snRNAseq data and the deconvoluted spatial transcriptomic data.^17^ These results were then compared between datasets and visualized on tissue sections.

### Statistical analysis:

DE analysis was performed using a negative binomial generalized linear model. Log2 fold-change of each gene comparison was calculated and plotted against its −log10(p-value) using a volcano plot. Genes with an absolute log2 fold-change greater than 1 and a significant p-value were considered significant and marked as either up or downregulated, depending on their log2 fold-change value. Spearman correlation analysis was used to compare L-R pairs that were downregulated in both, snRNAseq and spatial. A p-value < 0.05 was used to determine statistical significance. Seeds were set to allow for reproducibility. All statistical tests were carried out in R (version 4.2.2).

## RESULTS

### Part 1: Impact of different freezing techniques on spatial transcriptomic analysis of human liver samples.

**Figure 1:**
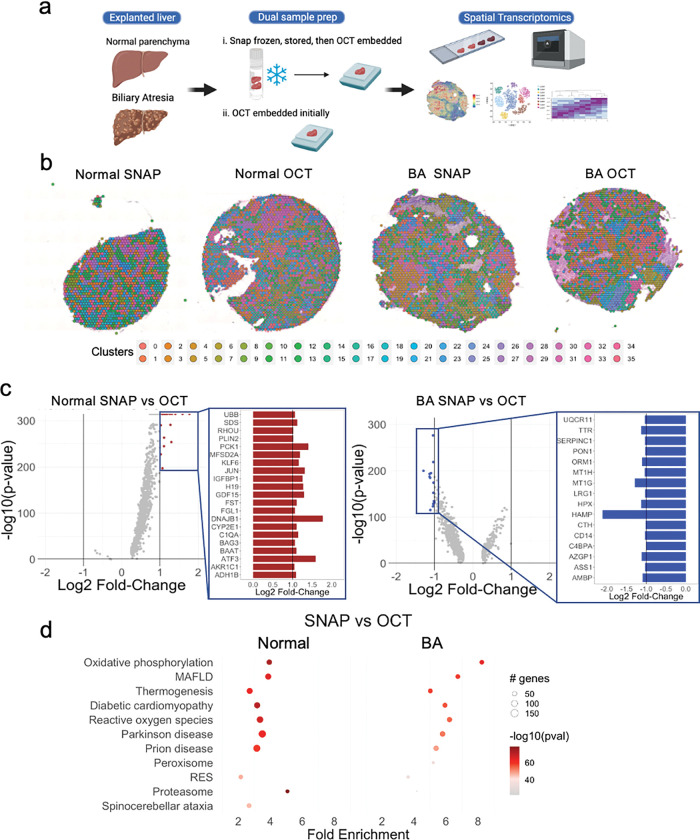
Liver tissue freezing technique impacts differential gene expression in oxidative stress pathways when using spatial transcriptomic analysis. (**a**) Samples of biliary atresia (BA) and normal parenchyma from hepatoblastoma liver were snap frozen, stored, embedded in OCT, and sectioned for ST (SNAP) or embedded in OCT and sectioned for ST (OCT). (**b**) Clustering of Visium spatial transcriptomics 55uM spots. (**c**) Volcano plots of SNAP vs OCT gene expression in normal liver and BAwith advanced fibrosis. Upregulated DE genes with a log2 fold-change greater than 1 are marked in red. Downregulated DE genes with a log2 fold-change less than −1 are marked in blue. Highlighted are the 21 upregulated genes in the normal liver and the 16 downregulated genes in the BA liver with advanced fibrosis. (**d**) Pathway analysis comparing SNAP and OCT preparation techniques in normal and BA with advanced fibrosis. MAFLD = metabolic associated fatty liver disease. RES = retrograde endocannabinoid signaling. Figure 1a was created using Biorender.

The constraints of clinical workflows, including the timing of biopsies and surgical procedures, can impact the ability to perform experiments using fresh liver samples. Also, many biorepositories bank samples of different disease states for future studies. In both instances, liver tissue is often snap frozen and stored at −80 C or in liquid nitrogen for future experiments.^18^ The Visium platform enables spatial transcriptomic analysis of frozen tissue specimens, and the manufacturer recommends samples be frozen using OCT at the time of collection. Based on our prior work with scRNAseq of human liver, we hypothesized that liver tissue that was snap frozen and stored would generate different transcriptomic profiles than liver tissue that was initially frozen using OCT.^19^ Fresh samples of normal liver tissue and BA with advanced fibrosis were collected and subject to two differing tissue preparation techniques at the time of collection: (i) snap freezing with storage in liquid nitrogen, followed by embedding in OCT at the time of retrieval from the freezer for transcriptomic analysis (SNAP), versus (ii) embedding in OCT up front with storage in liquid nitrogen (OCT) (Fig. 1a). A spatial map of gene expression for each 55 μm spot was generated for each sample, and clustering algorithms were utilized to identify groups of spots with similar gene expression patterns (Fig. 1b). The analysis resulted in a diverse set of 36 clusters of spots representative of the spatial complexity in the liver in both SNAP and OCT conditions. Next, DE analysis was performed to compare SNAP and OCT preservation techniques in normal and BA with advanced fibrosis samples. When comparing the two freezing techniques in normal liver, DE genes were largely upregulated, including activating transcription factor 3 (ATF3), DnaJ heat shock protein family member B1 (DNAJB1), and phosphoenolpyruvate carboxykinase 1 (PCK1) (fold change > 2.5) in SNAP relative to OCT (Fig. 1c). When comparing BA with advanced fibrosis between freezing conditions (SNAP vs. OCT), the subset of genes that were DE were primarily downregulated, including hepcidin antimicrobial peptide (HAMP) and metallothionein 1G (MT1G) (fold change < −2.2) in SNAP compared to OCT (Fig. 1c). We next performed pathway analysis of DE genes, highlighting effects on the proteasome, oxidative phosphorylation, and reactive oxygen species in SNAP relative to OCT in both normal and BA samples, indicating that preservation technique likely impacts downstream transcript quality in these pathways (Fig. 1d). In BA with advanced fibrosis, genes associated with oxidative phosphorylation, metabolic associated fatty liver disease (MAFLD)^20^, and thermogenesis had even larger fold changes in SNAP relative to OCT than normal livers, indicating that diseased tissue with advanced fibrosis may be more sensitive to tissue processing techniques (Fig. 1d). Given these findings, only the OCT samples were used for further analysis.

### Part 2: Integration of snRNAseq and spatial transcriptomic data via deconvolution enables the creation of a spatially resolved single-cell atlas of the human liver.

**Figure 2:**
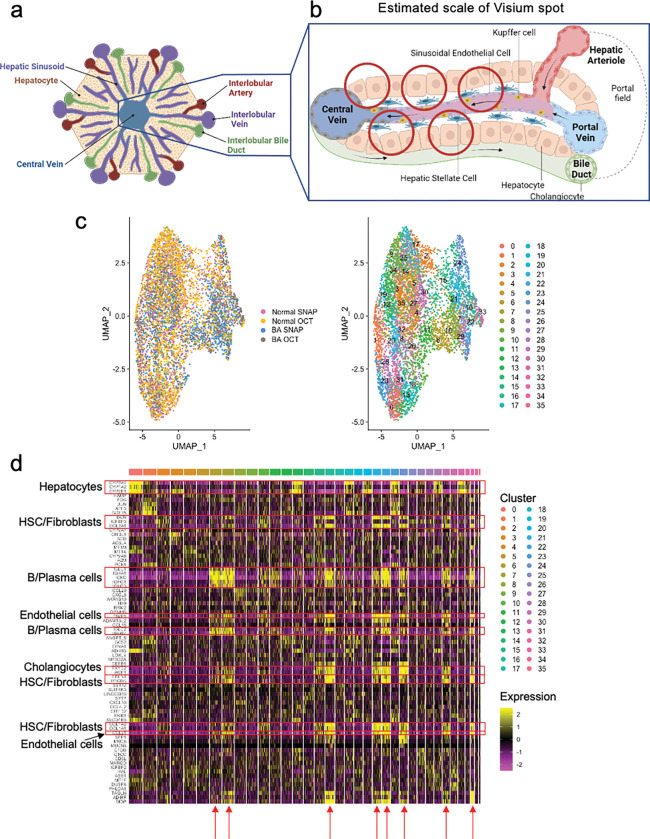
Clustering of spatial transcriptomic data spots yields mixed cellular phenotypes in human liver tissue. (**a**) Spatial organization of the liver portal tract showing individual cell types. (**b**) Liver sinusoid, with red circles corresponding to relative size of Visium spots (55μm). Given the spatial organization of the liver with immune cells and cholangiocytes scattered throughout, a single spot is unlikely to resolve discrete cell populations. (**c**) UMAP visualization of spatial spots after integration of data obtained from all samples, labelled by sample on the left and by cluster on the right. (**d**) Differential gene expression within spot clusters. Red arrows highlight clusters with a mix in phenotype (Endothelial cells, HSC/fibroblasts, cholangiocytes and immune cells) affirming that clustering of these spatial spots cannot resolve individual cell populations. HSC = hepatic stellate cells. Figures 2a and 2b were created using Biorender.

**Figure 3:**
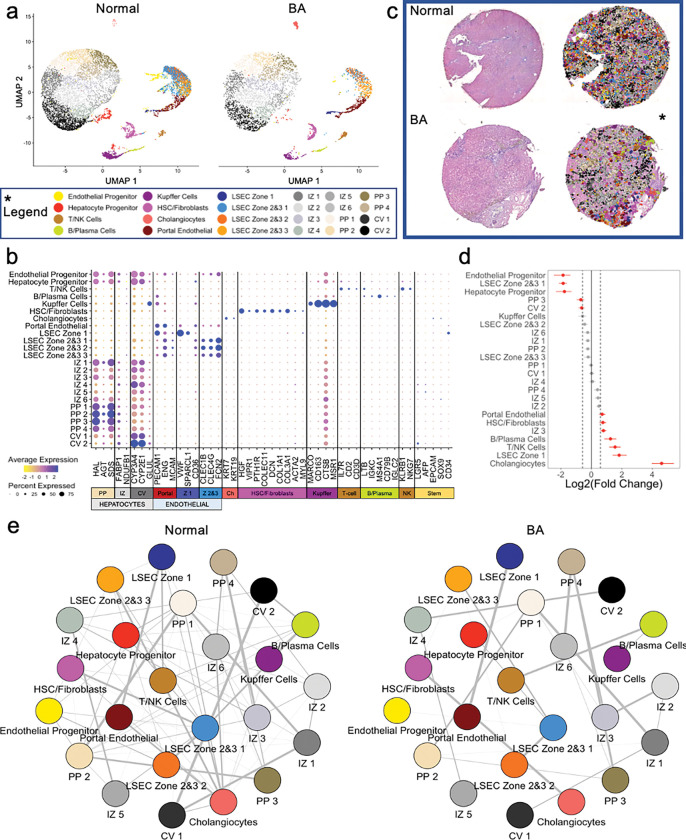
Deconvolution of spatial transcriptomic data using matched snRNAseq data enables the creation of a spatially resolved, single-cell transcriptomic atlas with 24 discrete liver cell phenotypes. (**a**) Paired snRNAseq data were generated for each Visium sample (normal liver and BA liver with advanced fibrosis). UMAP visualization of single cells clusters by sample after cell labelling using established markers. (**b**) Gene expression heatmap of individual cell types with corresponding cell-type markers. (**c**) Spatial visualization of deconvoluted spatial dataset. The left column represents the tissue slide after H&E principal staining and the right column includes overlay of single cell types after deconvolution. (**d**) Cell proportion comparison between normal and BA with advanced fibrosis using permutation testing (red signifies FDR < 0.05 and abs(Log2(Fold Change)) > 0.58). BA liver with advanced fibrosis has higher proportions of cholangiocytes, LSEC zone 1, T/NK cells, B/Plasma cells, IZ 3, HSC/Fibroblasts, and portal endothelial cells. Normal liver has higher proportions of endothelial progenitor cells, LSEC Zone 2&3 1, hepatocyte progenitor cells, PP 3 cells, and CV2 cells. (**e**) Correlation plots of cell clusters for both normal and BA with advanced fibrosis samples using spatially deconvoluted data. Line thickness correlates to spatial associations between two cell types. Strength of spatial association is decreased in BA with advanced fibrosis. CV = central-venous hepatocytes. HSC = hepatic stellate cells. IZ = interzonal hepatocytes. LSEC = liver sinusoidal endothelial cells. NK = natural killer cells. PP = periportal hepatocytes.

**Figure 4:**
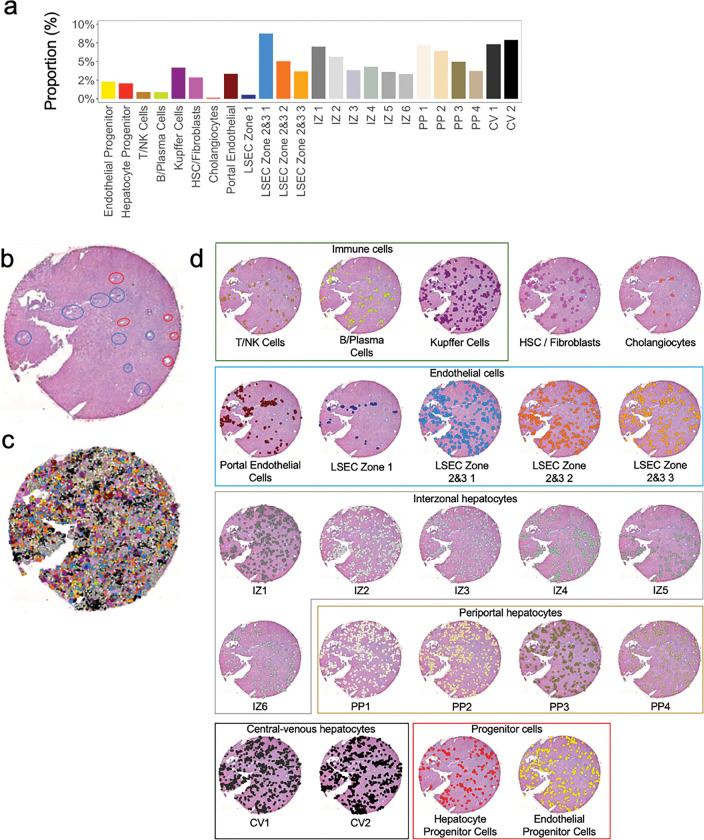
Deconvolution of spatial transcriptomic data enables identification of individual cell types in normal liver. (**a**) Cell proportion of each cluster in the snRNAseq sample. (**b**) H&E stained tissue section after annotation by a pathologist, with red circles corresponding to central veins and blue circles to periportal regions. (**c**) Spatial visualization of single-cell clustering after deconvolution overlayed on H&E stain. (**d**) Overlay of single-cell clustering after deconvolution visualized on H&E section for each cell type. CV = central-venous hepatocytes. HSC = hepatic stellate cells. IZ = interzonal hepatocytes. LSEC = liver sinusoidal endothelial cells. NK = natural killer cells. PP = periportal hepatocytes.

**Figure 5:**
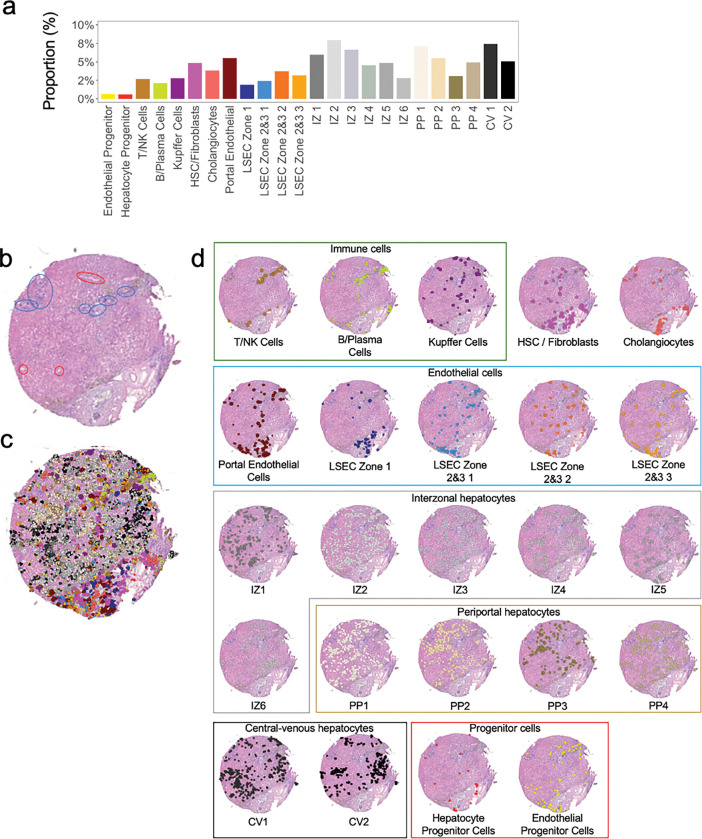
Deconvolution of spatial transcriptomic data enables identification individual cell types in BA with advanced fibrosis tissue. (**a**) Cell proportion of each cluster in the snRNAseq sample. (**b**) H&E stained tissue section after annotation by a pathologist, with red circles corresponding to central veins and blue circles to periportal regions. (**c**) Spatial visualization of single-cell clustering after deconvolution overlayed on H&E stain. (**d**) Overlay of single-cell clustering after deconvolution visualized on H&E section for each cell type. CV = central-venous hepatocytes. HSC = hepatic stellate cells. IZ = interzonal hepatocytes. LSEC = liver sinusoidal endothelial cells. NK = natural killer cells. PP = periportal hepatocytes.

Liver tissue is highly spatially organized with portal triads and central veins connected by sinusoids (Fig. 2a). The Visium spatial transcriptomic platform generates output ‘spots’ of 55μm diameter, which can represent 1–10 cell types per spot (Fig. 2b). When attempting to identify cell types based solely on Visium spots, multiple cell types are present in each spot, even when intentionally overclustering (Fig. 2c), as shown in the heatmap in Fig. 2d. Recent advances in informatics methods have been developed to map single cell transcriptomes from scRNAseq or snRNAseq outputs onto spatial transcriptomic datasets using a computational approach called ‘deconvolution’. There are two general approaches to deconvolution, which include identification of the percent of each cluster or cell type in a spatial spot (CARD^21^ or Seurat^10^) versus mapping a single cell type to a specific spot (Celltrek^15^). We implemented both deconvolution techniques, CARD and Celltrek (**Supplemental Fig. 1**). Given the complexity of liver tissue and the desire to understand spatial relationships between individual cell types and their transcriptomes, rather than just their location, we employed the second approach to map single cell types to Visium spots using Celltrek for downstream analysis since it improved output interpretability. To perform deconvolution, we first generated matched snRNAseq datasets from both normal and BA with advanced fibrosis samples. Unsupervised clustering was used to generate 24 clusters within the snRNASeq data, and cell lineage marker expression was used to annotate individual cell types (Fig. 3a,b). Relative shifts in cell proportions were evaluated between normal and BA with advanced fibrosis samples and revealed that BA tissue had increased cholangiocytes, liver sinusoidal endothelial cells (LSEC) zone 1, T/NK cells, B/Plasma cells, hepatic stellate cells (HSC)/fibroblasts, and portal endothelial cells (Fig. 3d). BA with advanced fibrosis had also decreased epithelial progenitor cells, LSEC zones 2&3, and hepatocyte progenitor cells. These annotated snRNAseq datasets were then used to deconvolute the spatial OCT samples (Fig. 3c). Each cell type was projected onto the spatial transcriptomic output and visualized relative to H&E staining to demonstrate density and spatial distribution in normal liver tissue (Fig. 4) and BA with advanced fibrosis tissue (Fig. 5). To increase our confidence in the deconvolution approach, we observed that the proportion of cells identified in each cell type cluster in deconvoluted data mirrored the snRNA seq data in both normal and BA liver tissues. In normal liver, the highest proportion of cells belonged to the LSEC zones 2&3 subcluster 1 cluster and the central-venous hepatocyte clusters (Fig. 4a). This is also seen in the visualization of the deconvoluted data on the tissue sample (Fig. 4c,d). Additionally, the annotated cells can be compared to pathologist-labeled periportal and central venous regions (Fig. 4b). While not spatially exclusive, cells identified in the portal endothelial cell gene expression cluster and the LSEC zone 1 fall in the periportal regions (Fig. 4d). In BA with advanced fibrosis, cells identified in the intrazonal 1 cluster were the largest proportion of cells (Fig. 5a), mirroring the distribution of cells in the deconvoluted data (Fig. 5c,d). When compared to microanatomy annotations (Fig. 5b), the immune cell types in BA were usually in periportal areas (Fig. 5d) whereas their spatial distribution was more diffuse in normal liver (Fig. 4d). Using these deconvoluted, spatially resolved single-cell data, colocalization analysis was completed to identify significant cellular interactions and avoidances (Fig. 3e). This revealed a loss of complexity in the cellular interactions in BA with advanced fibrosis, with immune populations interacting more with LSEC and hepatocyte populations in normal liver tissue.

### Part 3: Spatial transcriptomic data augments Ligand-Receptor Analysis in human livers.

**Figure 6:**
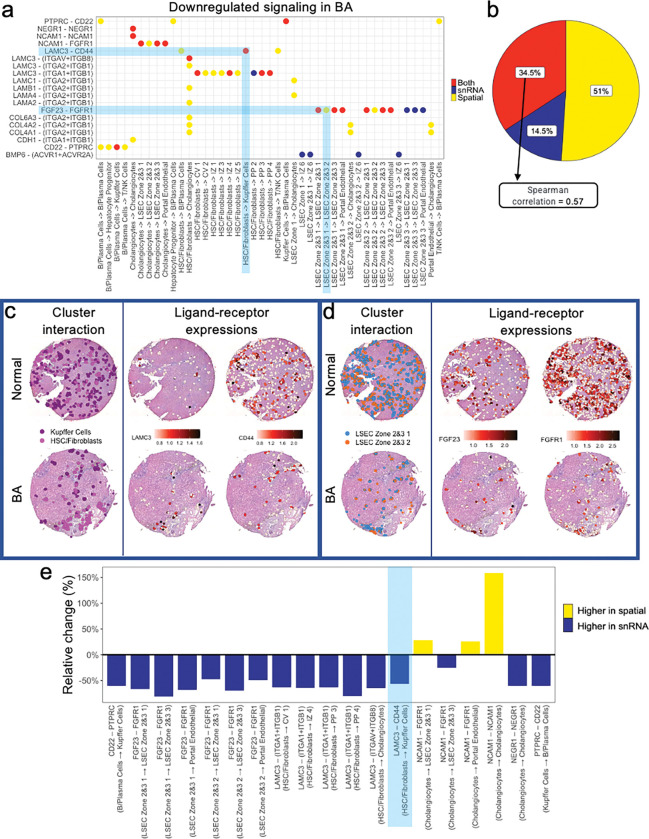
Cluster interaction analysis illustrates the difference in Ligand-Receptor Analysis in BA with advanced fibrosis when comparing snRNAseq and spatially deconvoluted transcriptomic data. (**a**) L-R pairs in interacting cell types show a decrease in signaling in BA liver with advanced fibrosis when compared to normal liver tissue. The three distinct colors represent whether interactions were only downregulated in snRNAseq data (blue), only in the spatially deconvoluted transcriptomic data (yellow), or in both datasets (red). Two representative cell-cell interactions are shaded in light blue. The first indicates downregulated L-R signaling only present in the deconvoluted spatial dataset (yellow dot, spatially visualized in panel **c**), and one indicates downregulated L-R signaling present in both datasets (red dot, spatially visualized in panel **d**). (**b**) Only 34.5% of downregulated L-R signaling pairs are present in both snRNAseq and spatial transcriptomic datasets (Spearman correlation = 0.57). 51% of downregulated L-R signaling pairs are only present in the spatial data, while 14.5% are only present in the snRNAseq data. (**e**) Relative change of communication strength in interacting cell types between deconvoluted spatial transcriptomic data and snRNAseq data. Signal is lower in the spatially deconvoluted data for most L-R signaling pairs. Highlighted in blue is the downregulated L-R signaling pair visualized in panel **d**. CV = central-venous hepatocytes. HSC = hepatic stellate cells. IZ = interzonal hepatocytes. LSEC = liver sinusoidal endothelial cells. NK = natural killer cells. PP = periportal hepatocytes.

Next, L-R analysis was performed, first on the snRNAseq data and then on the spatial transcriptomic data. Overall, the L-R pairs among interacting cell types showed a decrease in signaling in BA with advanced fibrosis when compared to normal liver, in either the snRNAseq data, spatial transcriptomic data, or both datasets (Fig. 6a). Overall, 51% of the L-R interactions were downregulated in only the spatial transcriptomic dataset, whereas 14.5% were downregulated only in the snRNAseq dataset with the remaining 34.5% downregulated in both (Fig. 6b). This highlights that the snRNAseq data alone are not sufficient to understand potential L-R interactions in a liver tissue sample.

Comparison with spatial deconvolution adds additional information regarding communication pathways. For example, the Laminin Subunit Gamma-3 (LAMC3)-CD44 interaction present between HSC/fibroblasts and Kupffer cells is downregulated in BA with advanced fibrosis in both the snRNAseq and spatial transcriptomic data (Fig. 6c). In this instance, spatial deconvolution analysis adds confidence to the results from the snRNAseq L-R analysis that BA with advanced fibrosis samples show potential interference with the communication pathway between these two cell types; specifically between LAMC3, a major component of basement membranes and CD44, a cell surface receptor that has been implicated in fibrotic and wound healing processes.^22–24^ In contrast, the Fibroblast Growth Factor-23 (FGF23) – Fibroblast Growth Factor Receptor-1 (FGFR1) interaction from LSEC zones 2&3 subcluster 1 to subcluster 2 is only downregulated in BA spatial transcriptomic data (Fig. 6d). For this interaction, the spatial transcriptomic data added information not previously seen when analyzing snRNAseq data. This suggests that within LSEC zones 2&3 of BA tissue, there is a distinctively spatial dysregulation of the FGF23-FGFR1 pathway, which has been implicated in both liver metabolism and the development of fibrosis.^25^

Ultimately, when comparing L-R interactions downregulated using both datasets, the relative change in communication strength in interacting cell groups between snRNAseq and spatially deconvoluted data was low, with a Spearman correlation of 0.57, suggesting that predicted L-R interactions from snRNAseq should be verified using deconvoluted spatial transcriptomic data (Fig. 6e).

## DISCUSSION

This study aimed to investigate the workflow of spatial transcriptomic analysis in clinical liver specimens, encompassing tissue preservation, data preprocessing, and downstream analysis. We first examined two different tissue processing techniques, snap-frozen in liquid nitrogen or cryopreserved in OCT blocks and observed that pre-analytic variables can impact spatial transcriptomic outputs. In both normal liver and BA with advanced fibrosis tissues, snap-frozen tissue was more likely to have alterations in cell stress pathways including oxidative phosphorylation, thermogenesis, and reactive oxygen species, which is consistent with our prior study integrating multiple liver scRNAseq datasets demonstrating that pathways involved in protein synthesis, cell death, and apoptosis signaling are affected by tissue processing.^19^ Our prior work and the current study highlight that the choice of experimental method does impact downstream single-cell transcriptomic analysis of clinical liver specimens. With regards to spatial transcriptomic outputs, OCT was superior to SNAP, as it did not upregulate cellular stress pathways to the same extent, thus preserving the underlying transcriptomics more accurately in both normal and fibrotic liver tissues.

Next, we explored how deconvolution using paired snRNAseq data enables the creation of a single-cell, spatially resolved transcriptomic dataset using banked clinical liver samples. Deconvolution is a crucial step in analyzing spatial transcriptomic datasets, as each spatial spot contains multiple cells and snRNAseq data lacks spatial information. We utilized snRNAseq data to define 24 different liver cell phenotypes and explored two popular deconvolution approaches: CARD^21^ and CellTrek^15^ (**Supplemental Fig. 1**). While CARD provided information on the percentage of each single nuclei cell type within each spot, visually interpreting this data with 24 cell phenotypes was challenging, making it difficult to confirm neighborhood relationships between cell types. Indeed, a recent publication examining BA liver used the Spotlight technique to generate a pie chart to visualize the proportions of cell types on each Visium spot, limiting analysis of potential interactions between cells of interest.^26^ In contrast, mapping snRNAseq data onto the Visium spots using CellTrek provided a more visually intuitive figure, enabling better validation of biological accuracy for specific cell types. Visual confirmation of deconvoluted cell types to pathologist-labeled liver tissue samples substantiated this approach. For instance, LSEC zone 1 cells were primarily in the pathologist-identified periportal regions, as expected (Figs. 3 and 4). In addition, deconvolution enabled additional downstream analysis. CellTrek specifically provided a spatial colocalization analysis that highlighted the spatial relationship between each cluster and cell type. This type of spatial analysis can be used for both validation of biological accuracy via the confirmation of known spatial relationships, as well as the discovery of new spatial relationships. Our analysis highlights how spatial transcriptomics can be used to validate potential cellular interactions derived from L-R analysis, a technique frequently used to explore scRNAseq and snRNAseq data. Although our number of samples is small, predicted L-R interactions from matched snRNAseq outputs poorly correlated with true spatial interactions using our deconvoluted spatial transcriptomic data in both health and fibrotic tissue (Spearman correlation = 0.57, Fig. 5). This suggests that investigators should be cautious when using L-R analysis to infer spatial relationships that may not be associated with pathologic processes in clinical samples.

While not the central focus of this study, our analysis revealed spatially relevant transcriptomic changes in BA with advanced fibrosis. First, BA tissue had a quantitative increase in immune cells, both T/NK cells and B/Plasma cells, as well as increased HSC/fibroblast cells. This is consistent with a prior study that used scRNAseq in BA, identifying a mixed immune and fibrotic response to damaged livers.^26^ The spatial distribution of these immune cells within the periportal area suggests that the immune cells are responding to injury within the portal triad in BA. This is also supported by the spatial co-localization analysis that demonstrated immune cells in BA interacted predominately with HSC/fibroblasts whereas in the normal liver tissue, they tended to interact more with LSEC and hepatocyte populations. Additionally, BA tissue showed a decrease in progenitor cells and LSEC zones 2&3. The decrease in epithelial and hepatocyte progenitor cells is expected with liver damage seen in end-stage BA. LSEC zones 2&3 cells are important spatially as zones 2&3 are histologically furthest from the portal triad and most susceptible to injury. The decreased proportion of LSEC cells suggests either that these cells are dying and replaced by fibrosis and/or that in the injury process, these cells can no longer be identified as LSEC zones 2&3 cells based on their gene expression profile. As Su et al. also demonstrated that LSEC zones 2&3 tend to retain their identities in snRNAseq cirrhosis data, it is likely that the decrease in cells is secondary to fibrotic development.^27^ In addition, these cells had a downregulation of L-R signaling from the LSEC zones 2&3 cells from subcluster 1 to subcluster 2 (Fig. 5). This suggests that there is a spatial dysregulation of cellular signaling within these zones, potentially contributing to unregulated fibrosis progression. Furthermore, there was a downregulation of the LAMC3-CD44 interaction from the HSC/Fibroblasts to the Kupffer cells in BA. This is interesting as other studies have highlighted CD44 in the inflammatory and fibrotic development of liver cirrhosis.^28,29^ While this finding may be due to the limitations of this study as discussed below, it is interesting to consider if previously known biomarker pathways of liver disease can be influenced by the addition of spatial analysis.

### Limitations

Various limitations should be considered in this study. First, its small sample size decreases the generalizability of the findings. Additionally, previous research has shown that the transcriptome is not equivalent to the proteome of the organism.^30^ In our transcriptomic based study, we cannot assume that the RNA analysis directly translates to the resulting phenotype of each liver cell. Finally, snRNAseq data are mapped onto spatial transcriptomic data based on the similarity between the gene profile of a given spatial spot and the gene profile of a single cell. The resulting labeled single-cell spot has the exact gene profile as the single-cell in the snRNAseq data. The accuracy of cell type labels and cell-cell communication used in the L-R analysis of the spatial data is therefore directly linked to the accuracy of deconvolution and cell labeling of the snRNAseq data.

### Conclusion

Spatial transcriptomics will continue to expand our understanding of clinical liver disease. In this study, we have highlighted important technical caveats and provided a roadmap for working with these complex datasets to explore the pathogenesis of end-stage BA at single-cell resolution. Using this approach, we observed that BA was characterized by a loss in cell-cell interaction complexity and dysregulation of FGF23-FGFR1 signaling and loss of LAMC3-CD44 interactions between hepatic stellate cells and Kupffer cells. Spatial deconvolution of single-cell transcriptomic data is a relatively new technique, and our study confirms that high-quality, single-cell resolution spatial transcriptomic techniques can be reliably employed to study both normal and diseased biobanked human liver samples.

## Data Availability

Data supporting the findings in this study have been made available on our lab’s Github (https://usctransplantlab.shinyapps.io/BAliver/). Raw primary data is available from the corresponding author upon reasonable request.
